# HuBMAPR: an R client for the HuBMAP data portal

**DOI:** 10.1093/bioadv/vbaf048

**Published:** 2025-03-10

**Authors:** Christine Hou, Shila Ghazanfar, Federico Marini, Martin Morgan, Stephanie C Hicks

**Affiliations:** Department of Biostatistics, Johns Hopkins Bloomberg School of Public Health, Baltimore, MD, 21205, United States; School of Mathematics and Statistics, The University of Sydney, Camperdown, NSW, 2006, Australia; Sydney Precision Data Science Centre, The University of Sydney, Camperdown, NSW, 2006, Australia; Charles Perkins Centre, The University of Sydney, Camperdown, NSW, 2006, Australia; Institute of Medical Biostatistics, Epidemiology and Informatics, University Medical Center Mainz, Mainz, 55118, Germany; Research Center for Immunotherapy (FZI), University Medical Center Mainz, Mainz, 55131, Germany; Roswell Park Comprehensive Cancer Center, Buffalo, NY, 14203, United States; Department of Biostatistics, Johns Hopkins Bloomberg School of Public Health, Baltimore, MD, 21205, United States; Department of Biomedical Engineering, Johns Hopkins University, Baltimore, MD, 21218, United States; Center for Computational Biology, Johns Hopkins University, Baltimore, MD, 21218, United States; Malone Center for Engineering in Healthcare, Johns Hopkins University, Baltimore, MD, 21218, United States

## Abstract

**Summary:**

The Human BioMolecular Atlas Program (HuBMAP) constructs the worldwide available platform to research the human body at the cellular level. The HuBMAP Data Portal encompasses a wide range of data resources measured on emerging experimental technologies at a spatial resolution. To broaden access to the HuBMAP Data Portal, we introduce an R client called HuBMAPR available on Bioconductor. This provides an efficient and programmatic interface that enables researchers to discover and retrieve HuBMAP data more easily and quickly.

**Availability and implementation:**

HuBMAPR is available at https://bioconductor.org/packages/HuBMAPR.

## 1 Introduction

The Human BioMolecular Atlas Program (HuBMAP), sponsored by the Common Fund at the National Institutes of Health, is dedicated to developing an open framework to map the human body at a single-cell resolution (HuBMAP Consortium, 2019). The HuBMAP Consortium comprises various research groups that are increasingly developing and using advanced computational tools to study the interaction, spatial organization, and specialization of trillions of cells within the adult human body. These efforts aim to understand how cellular structures contribute to organ and tissue function and how these processes relate to overall human health. HuBMAP supports this effort by hosting comprehensive data on the HuBMAP Data Portal (Jain *et al.*, 2023). Authorized contributors can upload their experimental data to the portal by adhering to the data submission guidelines (HuBMAP, 2025), thus expanding the shared resources available for scientific discovery.

HuBMAP data generally encompass five primary entity categories: (i) dataset, (ii) donor, (iii) sample, (iv) collection, and (v) publication. As of February 2025, more than 3000 datasets are available across 20 assay types (HuBMAP 2025), including Co-detection by indexing (Goltsev *et al.* 2018), Imaging Mass Cytometry (Chevrier *et al.* 2017, Chevrier *et al.* 2018, Rapsomaniki *et al.* 2018, Schulz *et al.* 2018), single-cell RNA Sequencing (Svensson *et al.* 2017), and Sequential Fluorescence *In-Situ* Hybridization (Eng *et al.*, 2019) (Supplementary Fig. S1), from more than 250 donors and more than 2300 samples across 31 different organs. HuBMAP datasets generated from related experiments (or sharing similar characteristics) are grouped into 18 HuBMAP collections. Despite these data resources being available on the HuBMAP Data Portal, currently, there is no programmatic interface in R (R Core Team 2024) to access, explore, retrieve and download these data. In this work, we address the problem by developing HuBMAPR Package R/Bioconductor (Gentleman *et al.* 2004) to allow researchers to explore and download HuBMAP data programmatically.

## 2 HuBMAPR R/bioconductor package

### 2.1 Overview of HuBMAP data portal and HuBMAPR

**Figure 1.**
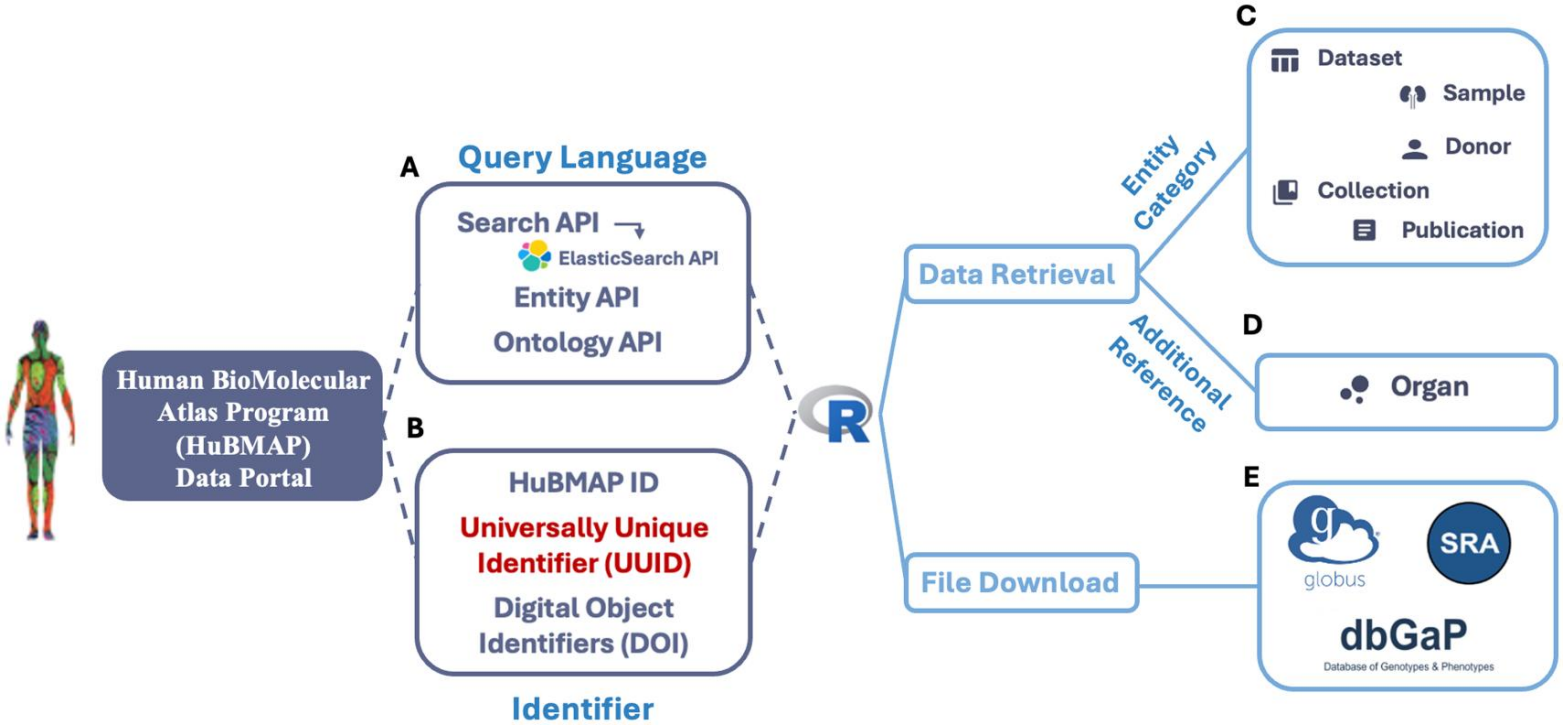
HuBMAPR builds a programmatic interface between the HuBMAP data portal (HuBMAP 2025) and the R programming language (R Core Team 2024) utilizing multiple APIs based on (A) a query language (HuBMAP Consortium 2019) and (B) extracting specific data entry based primarily on Universally Unique Identifiers (UUIDs). (C) Within R, the HuBMAPR package helps to explore and retrieve data from different entity categories. (D) The HuBMAPR package provides an additional reference for organ abbreviation and corresponding full name. (E) Data files can be accessed and downloaded via Globus (Globus 2010), NCBI Database of Genotypes and Phenotypes (dbGAP) (Tryka *et al.* 2014) or Sequence Read Archive (SRA) (Katz *et al.* 2022).

The HuBMAP Consortium offers several APIs (Jain *et al.* 2023) to ensure programmatic access to its components. The HuBMAPR package specifically integrates three APIs: Search API (Elastic 2025), Entity API, and Ontology API (Fig. 1A). Each API serves a distinct purpose with unique query capabilities, tailored to meet various needs. Using the packages httr2 (Wickham 2024) and rjsoncons (Morgan *et al.* 2024) packages, HuBMAPR effectively manages, modifies and executes multiple requests via these APIs, presenting responses in formats such as tibble or character. These outputs are further modified for clarity in the final results of the HuBMAPR functions. The Search API primarily searches for relevant data information and is referred to the Elasticsearch API (Jain *et al.* 2023). The Entity API is specifically utilized in the bulk_data_transfer() function for Globus URL retrieval, while the Ontology API is applied in the organ() function.

Using temporary storage to cache API responses facilitates efficient data retrieval by reducing the need for redundant requests to the HuBMAP Data Portal. This approach minimizes server load, improves response times (e.g. datasets() takes <4 s to retrieve more than 3500 datasets’ information), and enhances query efficiency. Periodically clearing cached data or directing it to a temporary directory ensures that the retrieved information remains relevant while managing storage effectively. This caching mechanism supports a smoother user experience when accessing data from the portal.

The HuBMAPR package retrieves data from the same five entity categories in HuBMAP using three different identifiers: (i) HuBMAP ID, (ii) Universally Unique Identifier (UUID), and (iii) Digital Object Identifiers. The HuBMAPR package primarily uses the UUID—a 32-digit hexadecimal number—and the more human-readable HuBMAP ID (Fig. 1B). Considering precision and compatibility with software implementation and data storage, the UUID serves as the primary identifier to retrieve data across various functions, with the UUID uniquely mapping to its corresponding HuBMAP ID. A systematic nomenclature is adopted for functions in the package by appending the entity category prefix to the concise description of the specific functionality. For example, dataset_detail() helps to retrieve the detailed metadata of one specific dataset, and donor_derived() provides the derived datasets of specific donors. Most of the functions are grouped by entity categories, simplifying the process of selecting the appropriate functions to retrieve the desired information associated with a UUID from the specific entity category. The structure of these functions is consistent across all entity categories, with some minor exceptions for collection and publication entities.

### 2.2 Data retrieval

The HuBMAPR package arranges HuBMAP data of each entity category chronologically by the last modification date, providing extensive physical, social, or ethnic demographic characteristics of the donor, including biological sex at birth, age, self-reported race, organ, body mass index, and other metadata. In addition, other features include experimental statistics such as analyte class, processing pipeline, sample category, affiliation information (e.g. contributor contacts, attribution group name, registration institution), and status updates such as publication date, publication status, and last modification date (HuBMAP Consortium, 2019). By carefully selecting and presenting these data details from the HuBMAP Data Portal, the HuBMAPR package offers a robust R-based interface for comprehensive data discovery, filtering, and extraction from datasets, samples, and donors (Fig. 1C); the package generates detailed textual descriptions, contributor lists, and links to related datasets for collections and publications, facilitating deeper insights into these resources (Fig. 1E). Furthermore, it serves as a reference tool for users seeking to identify organs associated with HuBMAP datasets, providing both organ abbreviations and their corresponding full names (Fig. 1C). This functionality enables users to efficiently filter and identify datasets relevant to specific organs, streamlining data exploration.

The HuBMAPR package facilitates the retrieval of the data provenance, including ancestors and descendants. The HuBMAP Data Portal defines an ancestor record as an individual record from which a specific donor, sample, or dataset is derived. In contrast, a descendant record is defined as an individual record derived from other preceding records. The donor initiates the provenance hierarchy, with the donor and the donor-derived sample organ considered foundational elements. Specific sample categories, such as section, suspension, and block, can be derived from the sample organ. Various assay types are applied to generate HuBMAP datasets from the samples. The provenance hierarchy culminates in the supporting dataset, particularly when the dataset is further processed by specific pipelines such as snapATAC (Fang *et al.* 2021), Salmon (Patro *et al.* 2017), or Cytokit (Czech *et al.* 2019). Corresponding functions can retrieve ancestor and descendant records for the donor, sample, and dataset. Due to the definition of the collection and publication entity, there is no function to retrieve ancestors and descendants.

### 2.3 Files delivery

The HuBMAP Data Portal offers access to open and restricted access to individual record files, adhering to the NIH Genomics Data Sharing Policy and other relevant legal frameworks. Public HuBMAP data can be accessed through Globus (2010), a secure and efficient cloud platform for large data storage and rapid file transfers (Fig. 1D). Using the unique dataset UUID, the HuBMAPR package connects to the HuBMAP public collection within the Globus research data management system, directing users to the Globus online website to preview and download raw data products, downstream analysis reports, metadata files, and visualizations.

The inability to programmatically discover, navigate, and transfer data files is a known limitation of HuBMAPR. We initially aimed to develop additional functions for the HuBMAPR package to enable users to transfer data files and directories locally and programmatically, avoiding manually navigating a browser, selecting files individually, and downloading them. However, these additional transfer functions introduced technical complexities without improving transfer speed or programmatic efficiency. To improve user accessibility, we proposed the experimental R package rglobus (Morgan 2024) as an alternative programmatic tool for data transfer. rglobus operates as a standalone R client, allowing users to discover and navigate collections and transfer files and directories across any Globus data folder. While rglobus is entirely independent of the HuBMAPR package and not specifically designed for the HuBMAP Data Portal, it can achieve the goal of transferring data files locally and programmatically, providing a versatile solution for users. Using HuBMAP data as the main example, the rglobus package documentation (Morgan 2024) included in the Supplementary Documents provides a detailed end-to-end pipeline to illustrate how the rglobus package operates on data transfer from Globus.

Restricted-access databases contain human-protected sequencing data that require special permissions. The NIH Data Access Committee manages access to restricted databases, and users must authenticate their identities to request downloads. Except for members of the HuBMAP Consortium, access to restricted-access databases can be granted through the Database of Genotypes and Phenotypes (dbGaP) (Tryka *et al.* 2014) or the Sequence Read Archive (SRA) (Katz *et al.* 2022), if available. However, the accessibility of restricted datasets depends on the availability of the dbGaP and SRA links or the sensitivity of the data. The bulk_data_transfer() function in the HuBMAPR package notifies users of the restricted status and provides appropriate feedback. If dbGaP and/or SRA links are available, the function generates error messages indicating restricted access and includes links to dbGaP and/or SRA. Users can copy and paste these links into a web browser, where additional instructions on these platforms guide them through the process of requesting permissions to access and download protected data files. In contrast, if neither the dbGaP nor the SRA links are available, suggesting that the data may contain highly sensitive information, the function generates a concise error message to inform users of the difficulty and the additional time and effort required to make these datasets accessible.

## 3 Conclusion

The HuBMAPR package serves as a robust tool for integrating and accessing comprehensive information from the HuBMAP Data Portal, effectively bridging the gap between the portal’s data and the specific needs of researchers. By providing a convenient and streamlined interface, the package allows users to explore data aligned with their research objectives. Researchers can filter various entity categories based on given conditions, such as age, BMI, gender, last modification date, and dataset type (e.g. sequencing data, image-based data, or experiment-processed data) that enable the identification of records with similar features or within approximate ranges. Once specific records are pinpointed, the functions allow retrieval of detailed information using UUIDs, including metadata, ancestors, and descendants. The package provides users with information on how to access or download data, indicating whether it is open-access or restricted. For restricted datasets, it guides users to relevant platforms such as dbGaP or SRA for requesting permissions. This functionality positions HuBMAPR as a faster and more efficient client for accessing data, offering extensive support, and facilitating complex research endeavors by providing related information critical for advanced scientific analysis.

## Supplementary Material

vbaf048_Supplementary_Data

## Data and availability

All published data are available on the HuBMAP Data Portal (HuBMAP 2025). The open-source software package HuBMAPR is based on the R programming language (R Core Team 2024), and the software license that we use is Artistic 2.0 (The Perl Foundation 2000–2006). This package is available in Bioconductor (at least version 3.20, https://bioconductor.org/packages/HuBMAPR) depending on R (at least version 4.4.0).
