# Supplementary material for: HuBMAPR: an R client for the HuBMAP data portal
Source: Bioinform Adv. 2025 Mar 10;5(1):vbaf048. doi: 10.1093/bioadv/vbaf048 (PMC11985162; doi:10.1093/bioadv/vbaf048)
Supplement: vbaf048_Supplementary_Data [file vbaf048_supplementary_data.zip › HuBMAPR_supplementary_material .pdf]

Supplementary Materials for "HuBMAPR": an R Client for the HuBMAP Data Portal

Christine Hou[1], Shila Ghazanfar[2,3,4], Federico Marini[5,6], Martin Morgan[7,*] and Stephanie C. Hicks[1,8,9,10,*]

[1]Department of Biostatistics, Johns Hopkins Bloomberg School of Public Health, Baltimore, MD, USA
[2]School of Mathematics and Statistics, The University of Sydney, Camperdown, New South Wales, AU
[3]Sydney Precision Data Science Centre, The University of Sydney, Camperdown, New South Wales, AU
[4]Charles Perkins Centre, The University of Sydney, Camperdown, New South Wales, AU
[5]Institute of Medical Biostatistics, Epidemiology and Informatics, University Medical Center Mainz, Mainz, Germany
[6]Research Center for Immunotherapy (FZI), University Medical Center Mainz, Mainz, Germany
[7]Roswell Park Comprehensive Cancer Center, Buffalo, NY, USA
[8]Department of Biomedical Engineering, Johns Hopkins University, Baltimore, MD, USA
[9]Center for Computational Biology, Johns Hopkins University, Baltimore, MD, USA
[10]Malone Center for Engineering in Healthcare, Johns Hopkins University, Baltimore, MD, USA
[*]Corresponding author. E-mail: shicks19@jhu.edu

Supplementary Figures

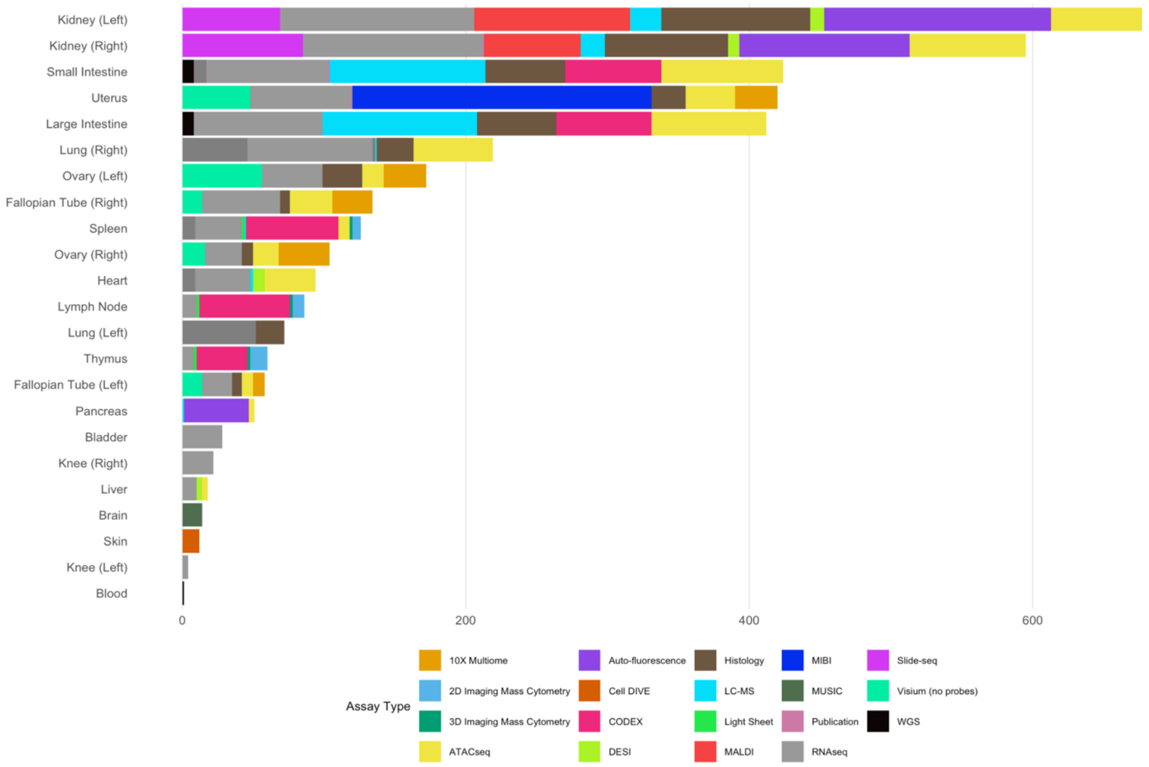

Supplementary Figure S1: Overview of tissue types in HuBMAP. HuBMAP Data Portal consists of the original datasets and corresponding support/processed datasets collected from a wide range of organs and processed by multiple assay types (as of February 2025)

Supplementary Documents

HuBMAPR 1.0.8

Explore Human BioMolecular Atlas Program Data Portal

Christine Hou

Department of Biostatistics, Johns Hopkins University
chris2018hou@gmail.com

Source: `vignettes/hubmapr_vignettes.Rmd`

Overview

'HuBMAP' data portal (https://portal.hubmapconsortium.org/) provides an open, global bio-molecular atlas of the human body at the cellular level. `HuBMAPR` package provides an alternative interface to explore the data via R.

The HuBMAP Consortium offers several APIs. To achieve the main objectives, `HuBMAPR` package specifically integrates three APIs:

- Search API: The **Search API** is primarily searching relevant data information and is referenced to the Elasticsearch API.
- Entity API: The **Entity API** is specifically utilized in the `bulk_data_transfer()` function for Globus URL retrieval
- Ontology API: The **Ontology API** is applied in the `organ()` function to provide additional information about the abbreviation and corresponding full name of each organ.

Each API serves a distinct purpose with unique query capabilities, tailored to meet various needs. Utilizing the `httr2` and `rjsoncons` packages, `HuBMAPR` effectively manages, modifies, and executes multiple requests via these APIs, presenting responses in formats such as tibble or character. These outputs are further modified for clarity in the final results from the `HuBMAPR` functions, and these functions help reflect the data information of HuBMAP Data Portal as much as possible.

HuBMAP Data Portal. This approach minimizes server load, improves response times (e.g. `datasets()` takes less than 4 seconds to retrieve more than 3500 records' information, shown below), and enhances overall query efficiency. By periodically clearing cached data or directing them to a temporary directory, the process ensures that the retrieved information remains relevant while managing storage effectively. This caching mechanism supports a smoother and more efficient user experience when accessing data from the portal.

HuBMAP Data incorporates three different identifiers:

- HuBMAP ID, e.g. HBM399.VCTL.353
- Universally Unique Identifier (UUID), e.g. 7036a70229eff1a51af965454dddbe7d
- Digital Object Identifiers (DOI), e.g. 10.35079/HBM399.VCTL.353.

The `HuBMAPR` package utilizes the UUID - a 32-digit hexadecimal number - and the more human-readable HuBMAP ID as two common identifiers in the retrieved results. Considering precision and compatibility with software implementation and data storage, UUID serves as the primary identifier to retrieve data across various functions, with the UUID mapping uniquely to its corresponding HuBMAP ID.

The systematic nomenclature is adopted for functions in the package by appending the entity category prefix to the concise description of the specific functionality. Most of the functions are grouped by entity categories, thereby simplifying the process of selecting the appropriate functions to retrieve the desired information associated with the given UUID from the specific entity category. The structure of these functions is heavily consistent across all entity categories with some exceptions for collection and publication.

Installation

`HuBMAPR` is a R package. The package can be installed by

```
if (!requireNamespace("BiocManager")) {
    install.packages("BiocManager")
```

HuBMAPR 1.0.8

Install development version from GitHub:

```
remotes::install_github("christinehou11/HuBMAPR")
```

Basic User Guide

Implementation Notes

This session is to guide on extending or customizing the `HuBMAPR` package to accommodate potential future changes in data structure, enhancing the package's long-term utility. We included a brief outline to illustrate the basics of the principles and approach to package design.

- Identify an API endpoint
- Provide an R client to translate R data structures to the arguments and parameters required by the API
- Handle the response consistently to argument and response validation
- Format the return value as a 'tibble' or 'character' to minimize cognitive demands on the user to interpret the result, and facilitate the incorporation into general R workflows

Load Necessary Packages

Load additional packages. `dplyr` package is widely used in this vignette to conduct data wrangling and specific information extraction.

```
library("dplyr")
library("tidyr")
library("ggplot2")
library("HuBMAPR")
library("pryr")
```

Data Discovery

`HuBMAP` data portal page displays chronologically (last modified date time) five categories of entity data:

- **Sample**
- **Donor**
- **Publication**
- **Collection**.

Using corresponding functions to explore entity data.

```
system.time({
    datasets_df <- datasets()
})
#>    user  system elapsed 
#>   0.801   0.032   2.384
object_size(datasets_df)
#> 1.07 MB

datasets_df
#> # A tibble: 3,811 × 14
#>    uuid        hubmap_id dataset_type dataset_type_additi
#>    <chr>       <chr>     <chr>        <chr>
#>  1 5f746bfbfd… HBM649.H… SNARE-seq2   ""
#>  2 93457f272f… HBM464.W… SNARE-seq2   ""
#>  3 84aa0347ea… HBM649.V… SNARE-seq2   ""
#>  4 6de9071380… HBM485.S… SNARE-seq2   ""
#>  5 3d3522198b… HBM982.B… SNARE-seq2   ""
#>  6 c6bc7e46a6… HBM483.G… SNARE-seq2   ""
#>  7 1bb771b1ff… HBM276.X… SNARE-seq2   ""
#>  8 822b2d0fa9… HBM724.H… SNARE-seq2   ""
#>  9 d372038ed1… HBM648.W… SNARE-seq2   ""
#> 10 f38b607f18… HBM588.W… SNARE-seq2   ""
#> # ℹ 3,801 more rows
#> # ℹ abbreviated name: ¹dataset_type_additional_informatio
#> # ℹ 8 more variables: sample_category <chr>, status <chr>
#> #   dataset_processing_category <chr>, pipeline <chr>, re
#> #   donor_hubmap_id <chr>, group_name <chr>, last_modifie
```

`samples()`, `donors()`, `collections()`, and `publications()` work same as above.

HuBMAPR 1.0.8

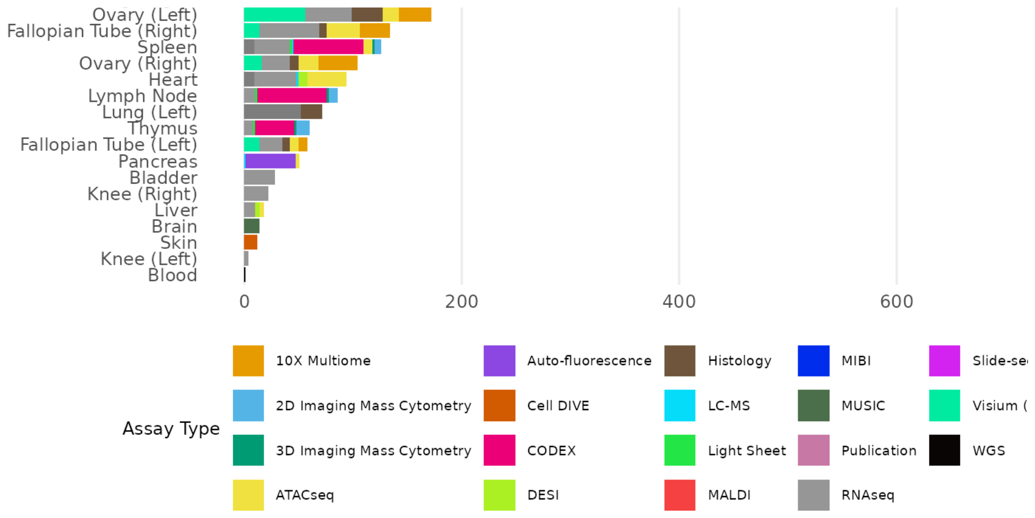

The default tibble produced by the corresponding entity function only reflects selected information. To see the names of selected information, use the following commands for each entity category. Specify the parameter of `as` to display information in the format of `"character"` or `"tibble"`.

```
# as = "tibble" (default)
datasets_col_tbl <- datasets_default_columns(as = "tibble")
datasets_col_tbl
#> # A tibble: 14 × 1
#>    columns
#>    <chr>
#>  1 uuid
#>  2 hubmap_id
#>  3 group_name
#>  4 dataset_type_additional_information
#>  5 dataset_type
#>  6 organ
#>  7 analyte_class
#>  8 dataset_processing_category
#>  9 sample_category
#> 10 registered_by
#> 11 status
#> 12 pipeline
#> 13 last_modified_timestamp
#> 14 donor_hubmap_id

# as = "character"
datasets_col_char <- datasets_default_columns(as = "characte
```

HuBMAPR 1.0.8

```
[illegible]
#>  [5] "dataset_type"                          "organ"
#>  [7] "analyte_class"                         "dataset_proce
#>  [9] "sample_category"                       "registered_by
#> [11] "status"                                "pipeline"
#> [13] "last_modified_timestamp"               "donor_hubmap_
```

`samples_default_columns()`, `donors_default_columns()`, `collections_default_columns()`, and `publications_default_columns()` work same as above.

A brief overview of selected information for five entity categories is:

```
tbl <- bind_cols(
    dataset = datasets_default_columns(as = "character"),
    sample = c(samples_default_columns(as = "character"), re
    donor = c(donors_default_columns(as = "character"), rep(
    collection = c(collections_default_columns(as = "charact
                   rep(NA, 10)),
    publication = c(publications_default_columns(as = "chara
                   rep(NA, 7))
)

tbl
#> # A tibble: 14 × 5
#>    dataset                            sample         donor
#>    <chr>                              <chr>          <chr>
#>  1 uuid                               uuid           hubm…
#>  2 hubmap_id                          hubmap_id      uuid
#>  3 group_name                         group_name     grou…
#>  4 dataset_type_additional_information sample_cate… Sex
#>  5 dataset_type                       organ          Age
#>  6 organ                              last_modifi… Body…
#>  7 analyte_class                      donor_hubma… Race
#>  8 dataset_processing_category        NA             last…
#>  9 sample_category                    NA             NA
#> 10 registered_by                      NA             NA
#> 11 status                             NA             NA
#> 12 pipeline                           NA             NA
#> 13 last_modified_timestamp            NA             NA
#> 14 donor_hubmap_id                    NA             NA
```

HuBMAPR 1.0.8

```
organs <- organ()
organs
#> # A tibble: 43 × 2
#>    abbreviation name
#>    <chr>        <chr>
#>  1 BD           Blood
#>  2 BL           Bladder
#>  3 BM           Bone Marrow
#>  4 BR           Brain
#>  5 BV           Blood Vasculature
#>  6 HT           Heart
#>  7 LA           Larynx
#>  8 LB           Bronchus (Left)
#>  9 LE           Eye (Left)
#> 10 LF           Fallopian Tube (Left)
#> # ℹ 33 more rows
```

Data Wrangling Examples

Data wrangling and filter are welcome to retrieve data based on interested information.

```
# Example from datasets()
datasets_df |>
    filter(organ == 'Small Intestine') |>
    count()
#> # A tibble: 1 × 1
#>       n
#>   <int>
#> 1   424
```

Any dataset, sample, donor, collection, and publication has a special **HuBMAP ID** and **UUID**, and **UUID** is the main ID to be used in most functions for specific detail retrievals.

The column of **donor_hubmap_id** is included in the retrieved tibbles from `samples()` and `datasets()`, which can help to join the tibble.

```
donors_df <- donors()
donor_sub <- donors_df |>
    filter(Sex == "Female",
```

HuBMAPR 1.0.8

```
             [illegible]
             last_modified_timestamp >= "2020-01-08" &
             last_modified_timestamp <= "2020-06-30") |>
    head(1)

# Datasets
donor_sub_dataset <- donor_sub |>
    left_join(datasets_df |>
                select(-c(group_name, last_modified_timestam
                rename("dataset_uuid" = "uuid",
                        "dataset_hubmap_id" = "hubmap_id"),
                by = c("hubmap_id" = "donor_hubmap_id"))

donor_sub_dataset
#> # A tibble: 0 × 19
#> # ℹ 19 variables: uuid <chr>, hubmap_id <chr>, group_name
#> #   Age <dbl>, Body Mass Index <dbl>, Race <chr>,
#> #   last_modified_timestamp <chr>, dataset_uuid <chr>, da
#> #   dataset_type <chr>, dataset_type_additional_informati
#> #   analyte_class <chr>, sample_category <chr>, status <c
#> #   dataset_processing_category <chr>, pipeline <chr>, re

# Samples
samples_df <- samples()
donor_sub_sample <- donor_sub |>
    left_join(samples_df |>
                select(-c(group_name, last_modified_timestam
                rename("sample_uuid" = "uuid",
                        "sample_hubmap_id" = "hubmap_id"),
                by = c("hubmap_id" = "donor_hubmap_id"))

donor_sub_sample
#> # A tibble: 0 × 12
#> # ℹ 12 variables: uuid <chr>, hubmap_id <chr>, group_name
#> #   Age <dbl>, Body Mass Index <dbl>, Race <chr>,
#> #   last_modified_timestamp <chr>, sample_uuid <chr>, sam
#> #   sample_category <chr>, organ <chr>
```

You can use `*_detail(uuid)` to retrieve all available information for any entry of any entity category given **UUID**. Use `select()` and `unnest_*()` functions to expand list-columns. It will be convenient to view tables with multiple columns but one row using `glimpse()`.

HuBMAPR 1.0.8

```
            organ == "Kidney (Right)") |>
    head(1) |>
    pull(uuid)

# Full Information
dataset_detail(dataset_uuid) |> glimpse()
#> Rows: 1
#> Columns: 35
#> $ ancestor_ids                      <list> <"de1076a42144c
#> $ ancestors                         <list> [["de1076a42144
#> $ contacts                          <list> [["Biomolecular
#> $ contains_human_genetic_sequences <lgl> FALSE
#> $ contributors                      <list> [["Biomolecular
#> $ created_by_user_displayname       <chr> "HuBMAP Process'
#> $ created_by_user_email             <chr> "hubmap@hubmapcc
#> $ created_timestamp                 <dbl> 1.711126e+12
#> $ creation_action                   <chr> "Create Dataset
#> $ data_access_level                 <chr> "public"
#> $ dataset_type                      <chr> "Auto-fluorescen
#> $ descendant_ids                    <list> "61530ed23518ee
#> $ descendants                       <list> [["Auto-fluores
#> $ description                       <chr> "Autofluorescenc
#> $ display_subtype                   <chr> "Auto-fluorescen
#> $ doi_url                           <chr> "https://doi.org
#> $ donor                             <list> ["Jamie Allen",
#> $ entity_type                       <chr> "Dataset"
#> $ group_name                        <chr> "Vanderbilt TMC'
#> $ group_uuid                        <chr> "73bb26e4-ed43-1
#> $ hubmap_id                         <chr> "HBM223.DJQM.264
#> $ immediate_ancestor_ids            <list> "de1076a42144de
#> $ immediate_descendant_ids          <list> "61530ed23518ee
#> $ index_version                     <chr> "3.6.1"
#> $ ingest_metadata                   <list> [[["a9099c6", '
#> $ last_modified_timestamp           <dbl> 1.71691e+12
#> $ metadata                          <list> ["Axio Scan.Z1'
#> $ origin_samples                    <list> [["Jamie Allen'
#> $ provider_info                     <chr> "VAN0042-RK-3 bl
#> $ published_timestamp               <dbl> 1.715267e+12
#> $ registered_doi                    <chr> "10.35079/HBM223
#> $ source_samples                    <list> [["Jamie Allen'
#> $ status                            <chr> "Published"
#> $ title                             <chr> "Auto-fluorescen
#> $ uuid                              <chr> "993bb1d6fa02e27
```

HuBMAPR 1.0.8

```
    select(contributors) |>
    unnest_longer(contributors) |>
    unnest_wider(everything())
#> # A tibble: 16 × 11
#>    affiliation                display_name email first_name
#>    <chr>                      <chr>        <chr> <chr>
#>  1 Biomolecular Multimodal… Jamie L. Al… jami… Jamie
#>  2 Delft Center for System… Lukasz Migas l.g.… Lukasz
#>  3 Biomolecular Multimodal… Nathan Heat… nath… Nathan
#>  4 Biomolecular Multimodal… Jeffrey M. … jeff… Jeffrey
#>  5 Delft Center for System… Leonor Tide… l.e.… Leonoor
#>  6 Delft Center for System… Raf Van de … Raf.… Raf
#>  7 Biomolecular Multimodal… Melissa A. … meli… Melissa
#>  8 Biomolecular Multimodal… Madeline E.… made… Madeline
#>  9 Biomolecular Multimodal… Ellie L. Pi… elli… Ellie
#> 10 Delft Center for System… Felipe Moser f.a.… Felipe
#> 11 Division of Nephrology … Mark deCaes… mark… Mark
#> 12 Division of Nephrology … Agnes B. Fo… agne… Agnes
#> 13 Division of Nephrology … Haichun Yang haic… Haichun
#> 14 Biomolecular Multimodal… Tina Tsui    tina… Tina
#> 15 Biomolecular Multimodal… Katerina V.… kate… Katerina
#> 16 Biomolecular Multimodal… Allison B. … alli… Allison
#> # ℹ 5 more variables: is_principal_investigator <chr>, la
#> #   metadata_schema_id <chr>, middle_name_or_initial <chr
```

`sample_detail()`, `donor_detail()`, `collection_detail()`, and `publication_detail()` work same as above.

Metadata

To retrieve the metadata for **Dataset**, **Sample**, and **Donor** metadata, use `dataset_metadata()`, `sample_metadata()`, and `donor_metadata()`.

```
dataset_metadata("993bb1d6fa02e2755fd69613bb9d6e08")
#> New names:
#> • `` -> `...1`
#> # A tibble: 22 × 2
#>    Key                             Value
#>    <chr>                           <chr>
#>  1 acquisition_instrument_model    "Axio Scan.Z1"
#>  2 acquisition_instrument_vendor   "Zeiss Microscopy"
#>  3 analyte_class                   "Endogenous fluoropho
#>  4 antibodies_path                 "extras/antibodies.ts
```

HuBMAPR 1.0.8

```
#>  7 [illegible]
#>  8 intended_tile_overlap_percentage ""
#>  9 is_image_preprocessing_required  "no"
#> 10 is_targeted                      "No"
#> # ℹ 12 more rows

sample_metadata("8ecdbdc3e2d04898e2563d666658b6a9")
#> # A tibble: 5 × 2
#>   Key                              Value
#>   <chr>                            <chr>
#> 1 donor.Age                        "71.0 years"
#> 2 donor.Apolipoprotein E phenotype "Apolipoprotein E pher
#> 3 donor.Pathology note             "Pathology note "
#> 4 donor.Race                       "White "
#> 5 donor.Sex                        "Male "

donor_metadata("b2c75c96558c18c9e13ba31629f541b6")
#> # A tibble: 8 × 2
#>   Key                Value
#>   <chr>              <chr>
#> 1 Age                "41.0 years"
#> 2 Body Mass Index    "37.10 kg/m2"
#> 3 Cause of Death     "Cerebrovascular accident "
#> 4 Death Event        "Natural causes "
#> 5 Mechanism of Injury "Intracranial hemorrhage "
#> 6 Race               "White "
#> 7 Sex                "Female "
#> 8 Social History     "Smoker "
```

Derived Data

Some datasets from the **Dataset** entity have derived (support) dataset(s). Use `dataset_derived()` to retrieve. A tibble with selected details will be retrieved as if the given dataset has a support dataset; otherwise, nothing returns.

```
# no derived/support dataset
dataset_uuid_1 <- "3acdb3ed962b2087fbe325514b098101"

dataset_derived(uuid = dataset_uuid_1)
#> NULL

# has derived/support dataset
```

HuBMAPR 1.0.8

```
#> Rows: 1
#> Columns: 6
#> $ uuid                    <chr> "bbbf5a5b29986dd57910daab
#> $ hubmap_id               <chr> ""
#> $ data_types              <chr> ""
#> $ dataset_type            <chr> "Histology [Image Pyramic
#> $ status                  <chr> ""
#> $ last_modified_timestamp <chr> "NA"
```

Sample and **Donor** have derived samples and datasets. In the `HuBAMPR` package, `sample_derived()` and `donor_derived()` functions are available to use to see the derived datasets and samples from one sample given sample UUID or one donor given donor UUID. Specify `entity_type` parameter to retrieve derived `Dataset` or `Sample`.

```
sample_uuid <- samples_df |>
    filter(last_modified_timestamp >= "2023-01-01" &
            last_modified_timestamp <= "2023-10-01",
            organ == "Kidney (Left)") |>
    head(1) |>
    pull(uuid)

sample_uuid
#> [1] "c40774aa2f52a2811db15c5ca1949314"

# Derived Datasets
sample_derived(uuid = sample_uuid, entity_type = "Dataset")
#> # A tibble: 12 × 2
#>    uuid                             derived_dataset_count
#>    <chr>                                            <int>
#>  1 4fddf6de0f42a7e2648b547affefc234                     1
#>  2 b6fd505b8e8e1829a2783570f9f25256                     0
#>  3 c3db2027e148e92fecb85e7d6a1fd708                     1
#>  4 3a10030d3323e5353cfdc3ada45cad86                     0
#>  5 71642e4c4a9cc12f59f3317b4a19adc9                     1
#>  6 bd42ab2f422e45ce6b0f3f55171de8aa                     0
#>  7 c8ad223f01b45b25e0dcb07c48a42762                     1
#>  8 f7b49444b974c98c6300e0bfe5fc3a75                     0
#>  9 beb1b65624fe85b527ee2ce80ef208b2                     1
#> 10 c25d6febe5b007ad32bc59246c99833d                     0
#> 11 744647801573d1d5700ee7523089734c                     1
```

```
# Derived Samples
sample_derived(uuid = sample_uuid, entity_type = "Sample")
#> # A tibble: 3 × 2
#>   uuid                             organ
#>   <chr>                            <chr>
#> 1 ec54b7d4ab4545166a0d121b3dc1ec3f Kidney (Left)
#> 2 ae98f6ca4f1f9950f7e7e1dedc2acc10 Kidney (Left)
#> 3 b099a37195f532e4b384020dc0e94bb5 Kidney (Left)
```

`donor_derived()` works same as above.

Provenance Data

For individual entries from **Dataset** and **Sample** entities, `uuid_provenance()` helps to retrieve the provenance of the entry as a list of characters (UUID, HuBMAP ID, and entity type) from the most recent ancestor to the furthest ancestor. There is no ancestor for Donor UUID, and an empty list will be returned.

```
# dataset provenance
dataset_uuid <- "3e4c568d9ce8df9d73b8cddcf8d0fec3"
uuid_provenance(dataset_uuid)
#> [[1]]
#> [1] "eba120ab7bbd864a6f6f3ad41e598d25, Sample"
#>
#> [[2]]
#> [1] "468d73d28b9e8c43ffa5fbd56d8e46e3, Sample"
#>
#> [[3]]
#> [1] "1c749716d32310351cb9557c7e2937a0, Sample"
#>
#> [[4]]
#> [1] "c09f875545a64694d70a28091ffbcf8b, Donor"

# sample provenance
sample_uuid <- "35e16f13caab262f446836f63cf4ad42"
uuid_provenance(sample_uuid)
#> [[1]]
#> [1] "0b43d8d0dbbc5e3923a8b963650ab8e3, Sample"
#>
#> [[2]]
#> [1] "eed96170f42554db84d97d1652bb23ef, Sample"
#>
#> [[3]]
```

HuBMAPR 1.0.8

```
# donor provenance
donor_uuid <- "0abacde2443881351ff6e9930a706c83"
uuid_provenance(donor_uuid)
#> list()
```

Related Data

Each **Collection** has related datasets, and use `collection_data()` to retrieve.

```
collections_df <- collections()
collection_uuid <- collections_df |>
    filter(last_modified_timestamp >= "2023-01-01") |>
    head(1) |>
    pull(uuid)

collection_data(collection_uuid)
#> # A tibble: 211 × 7
#>    uuid      hubmap_id dataset_type_additio…¹ dataset_type
#>    <chr>     <chr>     <chr>                  <chr>
#>  1 59c8093… HBM296.N… MIBI                   MIBI
#>  2 09dcc99… HBM374.N… MIBI                   MIBI
#>  3 c0ede74… HBM255.F… MIBI                   MIBI
#>  4 18a68d8… HBM297.D… MIBI                   MIBI
#>  5 4e6a2c1… HBM495.D… MIBI                   MIBI
#>  6 f5619b5… HBM452.K… MIBI                   MIBI
#>  7 2b56f0f… HBM887.D… MIBI                   MIBI
#>  8 54eec38… HBM439.H… MIBI                   MIBI
#>  9 b77be62… HBM225.P… MIBI                   MIBI
#> 10 36a80d4… HBM367.Q… MIBI                   MIBI
#> # ℹ 201 more rows
#> # ℹ abbreviated names: ¹dataset_type_additional_informat
#> #   ²last_modified_timestamp
#> # ℹ 2 more variables: status <chr>, organ <chr>
```

Each publication has related datasets, samples, and donors, and use `publication_data()` to see, while specifying `entity_type` parameter to retrieve derived `Dataset` or `Sample`.

```
publications_df <- publications()
publication_uuid <- publications_df |>
    filter(publication_venue == "Nature") |>
    head(1) |>
```

```
publication_data(publication_uuid, entity_type = "Dataset")
#> # A tibble: 1 × 2
#>   dataset_type uuid
#>   <chr>        <chr>
#> 1 CODEX        3a4fb23a49fba2647dba2b8f57976d25

publication_data(publication_uuid, entity_type = "Sample")
#> # A tibble: 3 × 2
#>   dataset_type uuid
#>   <chr>        <chr>
#> 1 Sample       5ded68dde822d1c03ee04fe58b978a7e
#> 2 Sample       ecca7757c924f2380669a15555d35a5d
#> 3 Sample       5d079766bf17544745c38d88d6923dbf
```

Additional Information

To read the textual description of one **Collection** or **Publication**, use `collection_information()` or `publication_information()` respectively.

```
collection_information(uuid = collection_uuid)
#> Title
#>   Spatiotemporal coordination at the maternal-fetal inter
#>  Description
#>   Beginning in the first trimester, fetally derived extra
#>  DOI
#>  -  https://doi.org/10.35079/hbm585.qpdv.454
#>  URL
#>  -  10.35079/hbm585.qpdv.454

publication_information(uuid = publication_uuid)
#> Title
#>  Organization of the human intestine at single-cell resol
#> Abstract
#>  We investigated the spatial arrangement of individual ce
#> Manuscript
#>  - Nature: https://doi.org/10.1038/s41586-023-05915-x
#> Corresponding Authors
#>  - John Hickey 0000-0001-9961-7673
#> Data Types
#>  - CODEX
#> Organs
#>  - Small Intestine
```

HuBMAPR 1.0.8

tion_contact() and collection_contributors() for **Collection** entity, or publication_authors() for **Publication** entity.

```
# Dataset
dataset_contributors(uuid = dataset_uuid)
#> # A tibble: 2 × 11
#>   affiliation                  display_name email first_name
#>   <chr>                        <chr>        <chr> <chr>
#> 1 "University of Californi… Xingzhao Wen xzwe… Xingzhao
#> 2 "University of Californi… Sheng Zhong  szho… Sheng
#> # ℹ 5 more variables: is_principal_investigator <chr>, la
#> #   metadata_schema_id <chr>, middle_name_or_initial <chr

# Collection
collection_contacts(uuid = collection_uuid)
#> # A tibble: 2 × 3
#>   name              affiliation
#>   <chr>             <chr>
#> 1 Shirley Greenbaum Department of Pathology, Stanford Uni
#> 2 Michael Angelo    Department of Pathology, Stanford Uni

collection_contributors(uuid = collection_uuid)
#> # A tibble: 13 × 3
#>    name               affiliation
#>    <chr>              <chr>
#>  1 Shirley Greenbaum Department of Pathology, Stanford Ur
#>  2 Inna Averbukh     Department of Pathology, Stanford Ur
#>  3 Erin Soon         Department of Pathology, Stanford Ur
#>  4 Gabrielle Rizzuto Department of Pathology, UCSF
#>  5 Noah Greenwald    Department of Pathology, Stanford Ur
#>  6 Marc Bosse        Department of Pathology, Stanford Ur
#>  7 Eleni G. Jaswa    Department of Obstetrics, Gynecology
#>  8 Zumana Khair      Department of Pathology, Stanford Ur
#>  9 David Van Valen   Division of Biology and Bioengineeri
#> 10 Leeat Keren       Department of Molecular Cell Biology
#> 11 Travis Hollmann   Department of Pathology, Memorial Sl
#> 12 Matt van de Rjin  Department of Pathology, Stanford Ur
#> 13 Michael Angelo    Department of Pathology, Stanford Ur

# Publication
publication_authors(uuid = publication_uuid)
#> # A tibble: 3 × 3
#>   name            affiliation          orcid_id
```

```
[illegible]
#> 3 Garry Nolan     Stanford University 0000-0002-8862-904[illegible]
```

File Transfer

For each dataset, there are corresponding data files. Most of the datasets' files are available on HuBMAP Globus with corresponding URLs. Some of the datasets' files are not available via Globus but can be accessed via dbGAP (database of Genotypes and Phenotypes) and/or SRA (Sequence Read Archive). However, some of the datasets' files are not available on any authorized platform.

Each dataset available on Globus has different components of data-related files to preview and download, including but not limited to images, metadata files, downstream analysis reports, raw data products, etc.

Use `bulk_data_transfer()` to know whether data files are open-accessed or restricted. Only open-accessed files can be downloaded for downstream analysis.

Files are publicly accessible

HuBMAP stored all public data files on Globus, which is an open-source and safe platform for large-size data storage. For every dataset in which the data files can be publicly accessed, the `bulk_data_transfer()` function will direct to the corresponding Globus webpage in Chrome.

```
uuid_globus <- "d1dcab2df80590d8cd8770948abaf976"

bulk_data_transfer(uuid_globus)
```

HuBMAPR 1.0.8

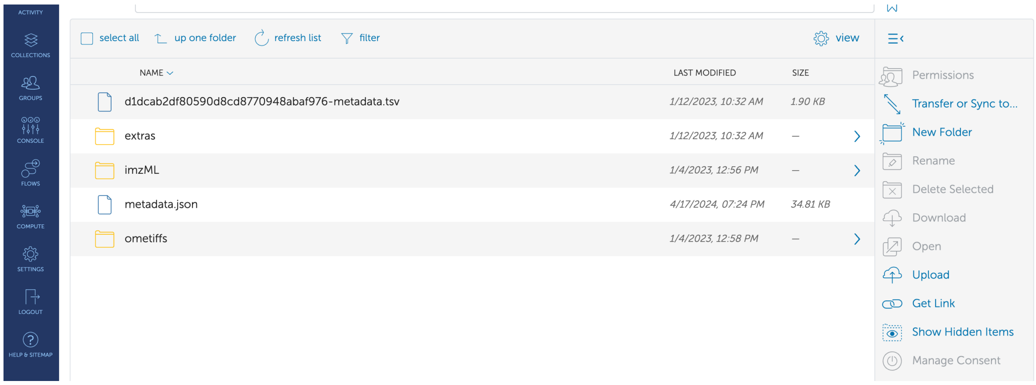

By selecting the data file and clicking on the “Download” button, the data file can be downloaded to the specific directory.

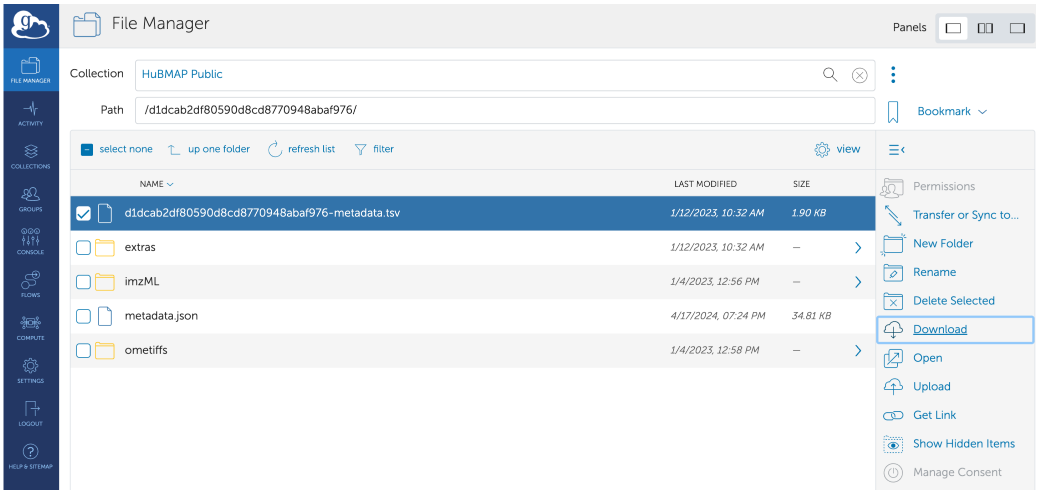

Alternative data transfer method using rglobus package

Martin Morgan, one of the `HuBMAPR` package creators, generated an experimental package called rglobus.

Globus is in part a cloud-based file transfer service, available at https://www.globus.org/. This package provides an *R* client with the ability to discover and navigate collections and to transfer files and directories between collections. Therefore, `rglobus` is an alternative method to transfer HuBMAP data files on the local computer using HuBMAP dataset UUID.

`rglobus` has the vignette documentation here using the HuBMAP collection as the main example to illustrate how to discover and navigate the correct collection and transfer the files.

Since `rglobus` is an experimental package, the functionality may not be complete. It is possible to see transfer issues while using functions.

Files are restricted

For every dataset in which the data files are restricted under dbGAP or SRA, the `bulk_data_transfer()` function will print out the instruction messages. The dbGaP and/or SRA link(s) allow the users to request the protected-access sequence data from an authenticated platform.

```
uuid_dbGAP_SRA <- "d926c41ac08f3c2ba5e61eec83e90b0c"

bulk_data_transfer(uuid_dbGAP_SRA)
```

```
Pruning cache
Error in bulk_data_transfer(uuid_dbGAP_SRA) :
This dataset contains protected-access human sequence data.
If you are not a Consortium member,
you must access these data through dbGaP if available.
dbGaP authentication is required for downloading.
View documentation on how to attain dbGaP access.
Additional Help: 'https://hubmapconsortium.org/contact-form/
Navigate to the 'Bioproject' or 'Sequencing Read Archive' li
dbGaP URL:
https://www.ncbi.nlm.nih.gov/projects/gap/cgi-bin/study.cgi?
Select the 'Run' link on the page to download the dataset.
Additional documentation: https://www.ncbi.nlm.nih.gov/sra/d
SRA URL: https://www.ncbi.nlm.nih.gov/sra/SRX13283313.)
```

Files are unavailable

For every dataset which the data files not available, the `bulk_data_transfer()` function will print out the messages.

```
uuid_not_avail <- "0eb5e457b4855ce28531bc97147196b6"

bulk_data_transfer(uuid_not_avail)
```

```
Pruning cache
Error in bulk_data_transfer(uuid_not_avail) :
This dataset contains protected-access human sequence data.
Data isn't yet available through dbGaP,
but will be available soon.
```

HuBMAPR 1.0.8

R session information

```
#> R version 4.4.2 (2024-10-31)
#> Platform: x86_64-pc-linux-gnu
#> Running under: Ubuntu 24.04.1 LTS
#>
#> Matrix products: default
#> BLAS:   /usr/lib/x86_64-linux-gnu/openblas-pthread/libbla
#> LAPACK: /usr/lib/x86_64-linux-gnu/openblas-pthread/libope
#>
#> locale:
#>  [1] LC_CTYPE=C.UTF-8       LC_NUMERIC=C           LC_TIM
#>  [6] LC_MESSAGES=C.UTF-8    LC_PAPER=C.UTF-8       LC_NAM
#> [11] LC_MEASUREMENT=C.UTF-8 LC_IDENTIFICATION=C
#>
#> time zone: UTC
#> tzcode source: system (glibc)
#>
#> attached base packages:
#> [1] stats     graphics  grDevices utils     datasets  met
#>
#> other attached packages:
#> [1] pryr_0.1.6       HuBMAPR_1.0.8    ggplot2_3.5.1    ti
#>
#> loaded via a namespace (and not attached):
#>  [1] utf8_1.2.4          rappdirs_0.3.3      sass_0.4.9
#>  [6] digest_0.6.37       magrittr_2.0.3      evaluate_1.0
#> [11] fastmap_1.2.0       lobstr_1.1.2        jsonlite_1.8
#> [16] purrr_1.0.4         scales_1.3.0        codetools_0.
#> [21] jquerylib_0.1.4     cli_3.6.3           rlang_1.1.5
#> [26] cachem_1.1.0        yaml_2.3.10         tools_4.4.2
#> [31] vctrs_0.6.5         rjsoncons_1.3.1     R6_2.5.1
#> [36] fs_1.6.5            ragg_1.3.3          pkgconfig_2.
#> [41] pillar_1.10.1       bslib_0.9.0         gtable_0.3.6
#> [46] systemfonts_1.2.1   xfun_0.50           tibble_3.2.1
#> [51] farver_2.1.2        htmltools_0.5.8.1   labeling_0.4
#> [56] prettyunits_1.2.0
```

On this page

HuBMAPR 1.0.8

[illegible]

Basic User Guide

File Transfer

R session information

Developed by Christine Hou, Martin Morgan, Federico Marini.

Site built with pkgdown 2.1.1.

rglobus 0.0.1.9000

Get Started

Martin Morgan

Source: vignettes/articles/a_get_started.Rmd

lifecycle experimental

Introduction & installation

This package is EXPERIMENTAL. It enables access to Globus collection management (including 'personal' collections on your own computer), including file and directory transfer. The functions implemented in this package are primarily from the Globus 'Transfer' API, documented at https://docs.globus.org/api/transfer/. Many other capabilities of Globus are not implemented.

Install the package if necessary

```
if (!requireNamespace("remotes", quiety = TRUE))
    install.packages("remotes", repos = "https://CRAN.R-proj
remotes::install_github("mtmorgan/rglobus")
```

Attach the package to your *R* session

```
library(rglobus)
```

Globus provides software to allow your laptop to appear as a collection. Follow the Globus Connect Personal installation instructions for your operating system, and launch the application. Then identify a location on your local disk to act as a collection.

On macOS, I created a directory `~/tmp/HuBMAP`, and then configured Globus Connect Personal to share that location. I did this by launching the application (it appears as an icon in the menu bar) then selecting 'Preferences' and using the '-' and '+' buttons to select the path to my local collection.

rglobus 0.0.1.9000

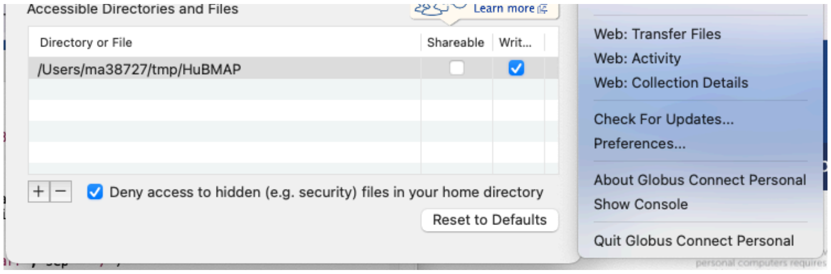

Discovering and navigating collections

The functions discussed here are based on the APIs described in [Endpoints and Collections](#) and [Endpoint and Collection Search](#).

Globus data sets are organized into collections. Start by discovering collections that contain the words "HuBMAP" and "Public", in any order.

```
hubmap_collections <- collections("HuBMAP Public")
hubmap_collections
## # A tibble: 4 × 2
##   display_name        id
##   <chr>               <chr>
## 1 HuBMAP Public       af603d86-eab9-4eec-bb1d-9d26556741b
## 2 HuBMAP Dev Public   2b82f085-1d50-4c93-897e-cd79d77481e
## 3 HuBMAP Stage Public 4b383482-8c5c-48fb-8b80-a450338ca38
## 4 HuBMAP Test Public  1e12c423-dd17-4095-a9f8-12555ee8334
```

Authentication

The first time `collections()` is invoked, Globus requires that you authenticate. A web page appears, where one can choose authentication via generic identifiers like ORCID or Google, or through an institution that you belong to. Follow the browser prompt(s) and return to *R* when done.

rglobus 0.0.1.9000

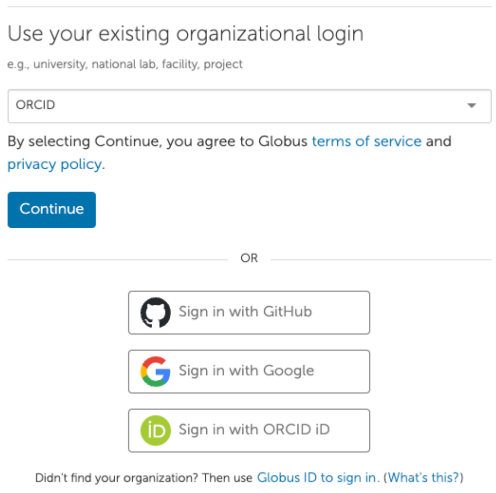

Collection content

Each collection is presented as directories and files. Focus on the 'HuBMAP Public' collection.

```
hubmap <-
    hubmap_collections |>
    dplyr::filter(display_name == "HuBMAP Public")
```

List the content of the collection.

```
globus_ls(hubmap)
## # A tibble: 2,308 × 4
##    name                             last_modified
##    <chr>                            <chr>
##  1 0008a49ac06f4afd886be81491a5a926 2024-07-26 13:35:21+0
##  2 0027cb59bcb4a34d5db83acaf934a9d9 2024-07-26 13:19:12+0
##  3 002e9747855eef1e69452b39713a7592 2024-08-13 19:04:13+0
##  4 00318be0b7cfa3c6ed7fbeab08fe700b 2024-07-26 12:58:42+0
##  5 004d4f157df4ba07356cd805131dfc04 2024-08-13 19:21:04+0
##  6 0066713ca95c03c52cb40f90ce8bbdb8 2024-04-17 23:24:41+0
##  7 007ae59344e7df0e398204ee40155cb0 2024-04-17 23:18:56+0
##  8 007f3dfaaa287d5c7c227651f61a9c5b 2024-07-26 14:46:55+0
##  9 00cc71c7e1cddac60e794044079faeee 2024-07-26 14:17:08+0
## 10 00d1a3623dac388773bc7780fcb42797 2024-04-17 23:02:27+0
## # ℹ 2,298 more rows
```

There are 2308 records, each corresponding to a HuBMAP dataset. Information about each dataset, e.g., the dataset with name

rglobus 0.0.1.9000

cover' the dataset name using the data portal or HuBMAPR, and then use `rglobus` to further explore the content).

List the content of a dataset of interest by adding a path to `globus_ls()`, e.g.,

```
path <- "0008a49ac06f4afd886be81491a5a926"
globus_ls(hubmap, path)
## # A tibble: 13 × 4
##    name                last_modified               size
##    <chr>               <chr>                      <int>
##  1 anndata-zarr        2021-11-21 09:40:50+00:00   4096
##  2 for-visualization   2021-11-21 09:41:41+00:00   4096
##  3 n5                  2021-11-21 09:35:26+00:00   4096
##  4 ometiff-pyramids    2021-11-21 09:35:26+00:00   4096
##  5 output_json         2021-11-21 09:39:13+00:00   4096
##  6 output_offsets      2021-11-21 09:37:00+00:00   4096
##  7 sprm_outputs        2021-11-21 09:15:36+00:00  12288
##  8 stitched            2021-11-21 04:44:53+00:00   4096
##  9 experiment.yaml     2021-11-21 04:32:41+00:00   1721
## 10 metadata.json       2024-07-26 13:35:21+00:00  90481
## 11 pipelineConfig.json 2021-11-21 04:58:21+00:00   5422
## 12 session.log         2021-11-21 09:40:55+00:00 18699854
## 13 symlinks.tar        2021-11-21 09:17:12+00:00  133120
```

The dataset consists of files and directories; further explore the content of individual directories by constructing the appropriate path.

```
path <- paste(path, "anndata-zarr", sep = "/")
globus_ls(hubmap, path)
## # A tibble: 1 × 4
##   name                                  last_modified
##   <chr>                                 <chr>
## 1 reg1_stitched_expressions-anndata.zarr 2021-11-21 09:40

path <- paste(path, "reg1_stitched_expressions-anndata.zarr"
hubmap |> globus_ls(path)
## # A tibble: 5 × 4
##   name   last_modified              size type
##   <chr>  <chr>                     <int> <chr>
## 1 X      2021-11-21 09:40:51+00:00  4096 dir
## 2 layers 2021-11-21 09:40:55+00:00  4096 dir
```

```
[illegible]
```

Note in the last example that the functions in `rglobus` are designed to support 'piping'.

Local collections

Collections owned by you appear in `collections()`, but the convenience function `my_collections()` provides another way to access these.

```
my_collections <- my_collections()
my_collections
## # A tibble: 1 × 2
##   display_name     id
##   <chr>            <chr>
## 1 Martin's HuBMAP 714ce2c4-3268-11ef-9629-453c3ae125a5
```

One aspect of the collection is that the path from the root (starting with `/`) or relative to the user home directory needs to be specified.

```
globus_ls(my_collections, "/Users/ma38727/tmp")
## # A tibble: 1 × 4
##   name   last_modified              size type
##   <chr>  <chr>                     <int> <chr>
## 1 HuBMAP 2024-08-14 21:09:38+00:00    96 dir

path <- "tmp"
globus_ls(my_collections, path)
## # A tibble: 1 × 4
##   name   last_modified              size type
##   <chr>  <chr>                     <int> <chr>
## 1 HuBMAP 2024-08-14 21:09:38+00:00    96 dir

## nothing here yet...
path <- "tmp/HuBMAP"
globus_ls(my_collections, path)
## # A tibble: 1 × 4
##   name                             last_modified
##   <chr>                            <chr>
## 1 d1dcab2df80590d8cd8770948abaf976 2024-08-14 21:09:38+00
```

rglobus 0.0.1.9000

enclosing folder.

```
mkdir(my_collections, "tmp/HuBMAP/test")
## # A tibble: 2 × 4
##   name                             last_modified
##   <chr>                            <chr>
## 1 d1dcab2df80590d8cd8770948abaf976 2024-08-14 21:09:38+0(
## 2 test                             2024-08-14 21:09:44+0(
```

Of course we could have used our operating system to create the directory in the path of the local collection.

Directory and file transfer

Directory and file transfer are described in the Task Submission API.

Start this section by ensuring we have the HuBMAP and our own collections

```
hubmap <-
    hubmap_collections |>
    dplyr::filter(display_name == "HuBMAP Public")
my_collections <- my_collections()
```

We illustrate directory and file transfer on a specific HuBMAP dataset. Here is the dataset and directory content

```
hubmap_dataset <- "d1dcab2df80590d8cd8770948abaf976"
globus_ls(hubmap, hubmap_dataset)
## # A tibble: 5 × 4
##   name                                          last_modi
##   <chr>                                         <chr>
## 1 extras                                        2023-01-1
## 2 imzML                                         2023-01-0
## 3 ometiffs                                      2023-01-0
## 4 d1dcab2df80590d8cd8770948abaf976-metadata.tsv 2023-01-1
## 5 metadata.json                                 2024-04-1
```

File transfer

in our local collection. Define the source and destination paths in the two collections.

```
source_path <-
    paste(hubmap_dataset, "metadata.json", sep = "/")
destination_path <-
    paste("tmp/HuBMAP/test", basename(source_path), sep = "/
```

Globus anticipates that large data transfers may be involved, so the `copy()` operation actually submits a task that runs asynchronously. Globus has confidence in the robustness of their file transfer, so adopt a 'fire and forget' philosophy – the task will eventually succeed or fail, perhaps overcoming intermittent network or other issues.

The `...` optional arguments to `copy()` are the same as the arguments for the lower-level `transfer()` function. By default, transfer tasks send email on completion, but we will check on task progress and respond appropriately. Each task can be labeled, the default label is provided by `transfer_label()`.

```
task <- copy(
    hubmap, my_collections,         # collections
    source_path, destination_path, # paths
    notify_on_succeeded = FALSE
)
task |>
    dplyr::glimpse()
## Rows: 1
## Columns: 3
## $ submission_id <chr> "88599a67-5a81-11ef-be9c-83cd94efb4
## $ task_id       <chr> "88599a66-5a81-11ef-be9c-83cd94efb4
## $ code          <chr> "Accepted"
```

We anticipate that `code` is "Accepted", indicating that the task is sufficiently well-formatted to be added to the task queue.

Task management

Use `task_status()` to check on status.

rglobus 0.0.1.9000

```
##   task_id                              type     status ni
##   <chr>                                <chr>    <chr>  <c
## 1 88599a66-5a81-11ef-be9c-83cd94efb466 TRANSFER ACTIVE Qu
```

The `status` column changes from `ACTIVE` to `SUCCEEDED` for successful tasks. An active task proceeding normally has `nice_status` either `Queued` or `OK`. An active task may be encountering errors, e.g., because the local connection is offline (`CONNECTION_FAILED`) or paused (`GC_PAUSED`) or that the source or destination file exists but the user does not have permission to read or write it (`PERMISSION_DENIED`). Perhaps unintuitively, Globus views these errors as transient (e.g., because the local collection may come back online) and so continues to try to complete the task. Active tasks that persist in an error state will eventually fail.

Failed tasks have status `FAILED`.

Let's write a simple loop to check on status, allowing the task to run for up to 60 seconds.

```
now <- Sys.time()
repeat {
    status <- task_status(task)$status
    complete <- status %in% c("SUCCEEDED", "FAILED")
    if (complete || Sys.time() - now > 60)
        break
    Sys.sleep(5)
}
```

If the task was successful, we should see the file in our local collection.

```
status
## [1] "SUCCEEDED"
globus_ls(my_collections, "tmp/HuBMAP/test")
## # A tibble: 1 × 4
##   name          last_modified              size type
##   <chr>         <chr>                     <int> <chr>
## 1 metadata.json 2024-08-14 21:09:48+00:00 34814 file
```

completed task generates a useful message without error.

```
task_cancel(task)
## TaskComplete: The task completed before the cancel reques
```

Directory transfer

Directory transfer is similar. Here we transfer the entire HuBMAP dataset to our local collection.

Specify the source path as the HuBMAP dataset, and the destination path as the dataset id in our local collection.

```
source_path <- hubmap_dataset
destination_path <- paste("tmp/HuBMAP", hubmap_dataset, sep
```

Submit the task as before, but add `recursive = TRUE` since this is a directory. As the task may take quite a while to complete, we will not change the default `notify_on_succeeded` option. Check on its initial status.

```
task <- copy(
    hubmap, my_collections,        # collections
    source_path, destination_path, # paths
    recursive = TRUE
)
task_status(task)
## # A tibble: 1 × 5
##   task_id                              type     status ni
##   <chr>                                <chr>    <chr>  <c
## 1 8c415b64-5a81-11ef-be9c-83cd94efb466 TRANSFER ACTIVE Qu
```

The task is added to a queue, and eventually the entire content of the HuBMAP dataset is transferred.

One can gain additional insight into the progress of the task by asking for `all_fields` of the task status. Relevant fields are the number of bytes transferred and the effective transfer rate. We use a helper function to format these values in a more intelligibly.

rglobus 0.0.1.9000

```
{
    ## use R's 'object_size' S3 class to pretty-print bytes
    x |>
        structure(class = "object_size") |>
        format(units = "auto")
}

task_status(task, all_fields = TRUE) |>
    dplyr::select(
        status, nice_status,
        bytes_transferred,
        effective_bytes_per_second
    ) |>
    dplyr::mutate(
        bytes_transferred = bytes_to_units(bytes_transferrec
        effective_bytes_per_second =
            bytes_to_units(effective_bytes_per_second)
    )
##   status nice_status bytes_transferred effective_bytes_pe
##   <chr>  <chr>       <chr>             <chr>
## 1 ACTIVE OK          227.8 Mb          486.9 Kb
```

The transfer can take some time, so in the interest of brevity we cancel the task.

```
task_cancel(task)
## Canceled: The task has been cancelled successfully.
```

Neglecting to set `recursive = TRUE` results in a `nice_status` `IS_A_DIRECTORY`. Globus nonetheless continues to try the file transfer, but we would recognize this as ‘user error’ and would cancel and re-submit the task.

```
task <- copy(
    hubmap, my_collections, # collections
    source_path,
    destination_path
)
## ...
task_status(task)
## # A tibble: 1 × 5
```

rglobus 0.0.1.9000

```
## [illegible]

## Oops, forgot the `recursive = TRUE` option
task_cancel(task)
task <- copy(
    hubmap, my_collections, # collections
    source_path,
    destination_path,
    recursive = TRUE
)
```

Complicated transfer tasks

Globus supports more complicated tasks, for instance submitting multiple transfer tasks at once, or synchronizing two locations. These will be developed in subsequent iterations of the package.

Session information

This vignette was compiled using the following software versions

```
sessionInfo()
## R version 4.4.1 Patched (2024-06-20 r86819)
## Platform: aarch64-apple-darwin23.5.0
## Running under: macOS Sonoma 14.5
##
## Matrix products: default
## BLAS:   /Users/ma38727/bin/R-4-4-branch/lib/libRblas.dyli
## LAPACK: /Users/ma38727/bin/R-4-4-branch/lib/libRlapack.dy
##
## locale:
## [1] en_US.UTF-8/en_US.UTF-8/en_US.UTF-8/C/en_US.UTF-8/en_
##
## time zone: America/New_York
## tzcode source: internal
##
## attached base packages:
## [1] stats     graphics  grDevices utils     datasets  met
##
## other attached packages:
## [1] rglobus_0.0.1
##
## loaded via a namespace (and not attached):
```

rglobus 0.0.1.9000

```
[illegible]
## [13] R6_2.5.1          rjsoncons_1.3.1   generics_0.1.3
## [17] httr2_1.0.2       knitr_1.48        htmlwidgets_1.6.
## [21] desc_1.4.3        openssl_2.2.0     bslib_0.7.0
## [25] rlang_1.1.4       utf8_1.2.4        cachem_1.1.0
## [29] xfun_0.46         fs_1.6.4          sass_0.4.9
## [33] withr_3.0.1       pkgdown_2.1.0     magrittr_2.0.3
## [37] askpass_1.2.0     rappdirs_0.3.3    lifecycle_1.0.4
## [41] evaluate_0.24.0   glue_1.7.0        whisker_0.4.1
## [45] fansi_1.0.6       rmarkdown_2.27    tools_4.4.1
## [49] htmltools_0.5.8.1
```

On this page

Introduction & installation

Discovering and navigating collections

Directory and file transfer

Session information
